# Thematic Analysis of Coroners' Prevention of Future Deaths (PFDs) Reports in Mental Health Related Suicide

**DOI:** 10.1192/bjo.2024.393

**Published:** 2024-08-01

**Authors:** Dean Manning, Shweta Mittal

**Affiliations:** 1 Nottinghamshire Healthcare NHS Foundation Trust, Nottingham, United Kingdom; 2 Sheffield Health and Social Care NHS Foundation Trust, Sheffield, United Kingdom

## Abstract

**Aims:**

To identify professional and organisational-related themes in Coroners' PFDs reports which contributed to mental health related suicide.

**Methods:**

We reviewed Coroners' PFDs reports via the UK Judiciary website. We filtered reports by those which were mental health related deaths and included the keyword ‘suicide’. 100 reports were reviewed starting with the most recent which was August 2023. We reviewed which Coroner's area the reports originated from and the age and gender of the deceased. Then, we examined the contents of the PFDs reports including the inquest conclusion, circumstances of death and concerns raised by the coroner. Themes were identified and grouped into patient-related, professional-related, and organisational-related factors that may have contributed to the death by suicide.

**Results:**

Reports were reviewed from across the UK. The highest number of reports were from the coroner area of Manchester South (12%).

From those reports whereby the deceased's age was mentioned, the mean age was 36 with an age range of 14–81 years (35% of reports did not include the deceased's age).

61% of reports were of males and 39% females.

The main professional-related factors identified from thematic analysis of the PFDs reports were issues around risk assessment and management (45%), lack of interprofessional communication and collaboration (33%), inadequate clinical queries/assessment (25%), lack of consultation of family/carers (17%) and lack of treatment/follow up plan following discharge (11%).

The main organisational-related factors were inadequate service provision for the population covered (20%), inadequate training/knowledge (18%), inadequate staffing or reliance on agency staff (15%), poor systems in place including information technology (13%) and lack of audit or evidence of learning from prior investigations & events (11%).

Patient-related factors were less commonly identified but included lack of engagement with services, denying suicidality and autistic spectrum disorder.

**Conclusion:**

The commonest theme was issues around risk assessment and management which was identified in 45% of suicides. It is hoped by highlighting common themes arising from PFDs reports across the UK this analysis could inform targeted improvements in practice that will lead to reductions in mental health related suicide which is the need of the hour.

